# Combining the Optimized Maximum Entropy Model to Detect Key Factors in the Occurrence of *Oedaleus decorus asiaticus* in the Typical Grasslands of Central and Eastern Inner Mongolia

**DOI:** 10.3390/insects15070488

**Published:** 2024-06-29

**Authors:** Xiaolong Ding, Bobo Du, Longhui Lu, Kejian Lin, Rina Sa, Yang Gao, Jing Guo, Ning Wang, Wenjiang Huang

**Affiliations:** 1 Institute of Grassland Research, Chinese Academy of Agricultural Sciences, Hohhot 010010, China; dxl15621119356@163.com (X.D.); dubobo@caas.cn (B.D.); linkejian@caas.cn (K.L.); 2 State Key Laboratory for Biology of Plant Diseases and Insect Pests, Hohhot 010010, China; 3 State Key Laboratory of Remote Sensing Science, Aerospace Information Research Institute, Chinese Academy of Sciences, Beijing 100094, China; lulh@aircas.ac.cn (L.L.); guojing211@mails.ucas.ac.cn (J.G.); 4 XilinGol Grassland National Nature Reserve Administration, XilinGol 026000, China; sarina179@126.com; 5 Inner Mongolia Academy of Forestry Sciences, Hohhot 010010, China; 15847176298@163.com

**Keywords:** grasshopper, typical steppe, MaxEnt, remote sensing

## Abstract

**Simple Summary:**

In China, *Oedaleus decorus asiaticus* is a typical pest that poses a serious threat to Inner Mongolian grasslands. However, the large-scale monitoring and control of grasshopper is still a priority that needs to be improved, so it is particularly important to identify environmental factors that influence locust breeding and growth to reveal the role of environmental factors in the occurrence of the dominant species *Oedaleus decorus asiaticus* in Inner Mongolia, and to study the potential spatial distribution of *Oedaleus decorus asiaticus* in typical grasslands. We matched the Maxent model with remote sensing, meteorological, and geographical data to identify environmental factors associated with *Oedaleus decorus asiaticus* occurrence and to identify possible grasshopper habitats. These efforts could effectively reduce the cost and time investment in grasshopper management.

**Abstract:**

Grasshoppers pose a significant threat to both natural grassland vegetation and crops. Therefore, comprehending the relationship between environmental factors and grasshopper occurrence is of paramount importance. This study integrated machine learning models (Maxent) using the kuenm package to screen MaxEnt models for grasshopper species selection, while simultaneously fitting remote sensing data of major grasshopper breeding areas in Inner Mongolia, China. It investigated the spatial distribution and key factors influencing the occurrence of typical grasshopper species in grassland ecosystems. The modelling results indicate that a typical steppe has a larger suitable area. The soil type, above biomass, altitude, and temperature, predominantly determine the grasshopper occurrence in typical steppes. This study explicitly delineates the disparate impacts of key environmental factors (meteorology, vegetation, soil, and topography) on grasshopper occurrence in typical steppes. Furthermore, it provides a methodology to guide early warning and precautions for grasshopper pest prevention. The findings of this study will be instrumental in formulating future management measures to guarantee grass ecological environment security and the sustainable development of grassland.

## 1. Introduction

China contains the world’s largest terrestrial ecosystem, with nearly 400 million hectares of natural grasslands. The grasshopper is a major pest in Inner Mongolia, causing significant damage both to Chinese animal husbandry and the ecology of the grasslands, as well as posing a serious threat to the production and survival of local farmers and herders [1,2,3,4]. Remote sensing technology allows the tracking of grasshoppers in real-time, allowing control measures to be implemented ahead of time to reduce losses [5,6].

Since the 1980s, information derived from remote sensing data have been widely used for grasshopper management, contributing to better and more effective control of locust outbreaks and plagues worldwide insects [7]. Nevertheless, despite technological advancements and improvements in monitoring and control, locust outbreaks continue to cause devastation and hunger. One reason is ineffective monitoring, management or population control in some grasshopper habitats, for example, due to a lack of available resources and technology [7]. One of the most crucial challenges for implementing cost- and time-effective pest control is the identification of grasshopper habitats and potential breeding locations [8].

Grasshoppers are associated with specific habitats, characterized by a variety of factors that facilitate the survival of the insects within both time and space. Many factors restrict the geographical distribution of grasshoppers [9]. These mainly include terrain, climate, soil, and vegetation, while hydrology, natural enemies, and human factors also have some effect. The presence and spread of grasshoppers are obviously influenced by the type, composition, and growth characteristics of the vegetation, especially herbaceous plants, which are the primary food source for grasshoppers [10,11,12]. Specifically, the variety and makeup of the vegetation impact the way that grasshoppers feed, and the growth of the vegetation affects the hydrothermal conditions of the area near the surface, which in turn affects the grasshoppers’ ability to survive and thrive [11,13,14]. The influence of soil on the growth and breeding of grasshoppers is primarily determined by the soil temperature, texture, moisture content, and salt content. There are obvious differences in the physical and chemical properties of different types of soil. The restricted surface environment defined by soil temperature and humidity has a clear influence on grasshopper activity [12,13,15]. During the spawning period, females prefer to lay their eggs in hard, dry soil; thus, the firmness of the soil resulting from both the soil texture and moisture content during the spawning period also has a significant impact on grasshoppers [15].Typically, low vegetation cover promotes grasshopper spawning, while high vegetation cover hinders this process [4,16]. Temperature and precipitation differences caused by altitude also have a significant impact on grasshopper populations; this is seen especially in areas where temperatures are lower due to altitude, affecting both the growth and development of the grasshoppers [17,18]. The presence of grasshoppers is closely related to the characteristics of the vegetation. An excessive amount of vegetation prevents sunlight from reaching the ground, which lowers the temperature and restricts grasshopper activity [19]. Grasshopper spawning is affected by the presence of bare terrain, and a lack of sufficient food sources and adequate protection provided by low vegetative cover also compromises grasshopper survival. In the grasslands of Inner Mongolia, overly damp soil adversely affects eggs during the spawning and overwintering stages, whereas excessive dryness compromises embryonic development. Too much soil moisture will cause grasshopper eggs to freeze or ice before the freezing period, which will increase the likelihood that they will die. Mildew is easily formed in warm, moist soil during the spawning stage, while, in contrast, dryness favors the overwintering of eggs. The opposite is true during the incubation stage [20,21,22,23,24]. Based on the findings of studies on factors influencing the grasshopper lifecycle, the integration of parameters such as rainfall, temperature, soil type, vegetation coverage, vegetation type, and altitude can be utilized to construct a suitability index.

Species distribution models (SDMs) are often used to predict and estimate the geographical range of species, and when the presence of a species in a given location is recorded, the combination of environmental factors associated with the species allows the occurrence probability or suitability of the target species to be estimated. In SDMs, MaxEnt models can be used to evaluate and predict the distribution of species by finding the maximum entropy of species distribution probabilities from known species distribution data and environmental factors. MaxEnt models have been used to assess the distribution of endangered species, the extent of the presence of protected animals, and to predict the geographical distribution of invasive species [25,26,27,28]. However, there is very little literature on the application of the MaxEnt model to insects from the time the model was first developed to the present. Currently, the MaxEnt model performs best among the SDMs in terms of the species presence records fitting environmental factors to assess suitability [29].

This study used a combination of the MaxEnt model and remote sensing data to conduct a species–environmental matching model. The specific objectives were (1) to use the MaxEnt model to construct a model defining the relationship between grasshopper breeding sites and environmental factors in the typical grassland regions of central and eastern Inner Mongolia, (2) to analyze the environmental variables most closely linked to grasshopper outbreaks and determine the factors most applicable to the typical grasslands of central and eastern Inner Mongolia, and (3) to study the associations between critical growth factors and grasshopper occurrence in the typical grasslands of central and eastern Inner Mongolia.

## 2. Materials and Methods

### 2.1. Study Area

**Figure 1:**
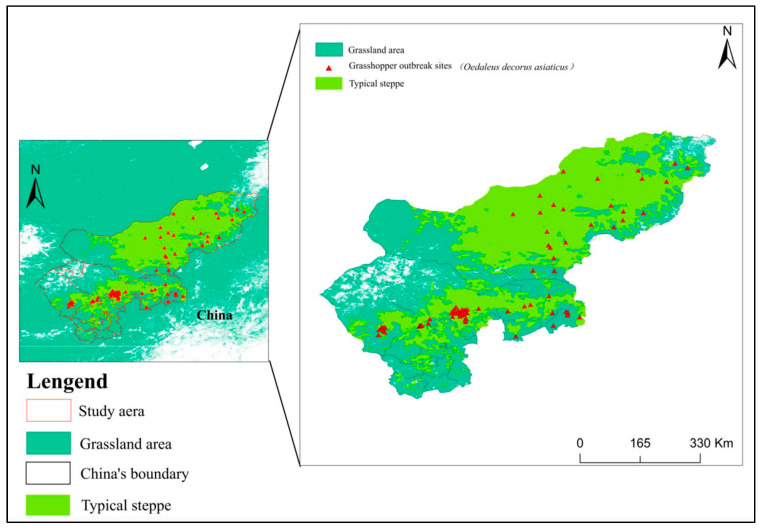
Location of the study area.

Typical grassland is the main natural grassland in Inner Mongolia, and the east-central region of Inner Mongolia is the largest area of typical grassland in China with the widest distribution [1]. The Xilingol plateau constitutes the center of the distribution of this grassland, which includes significant portions of Xilinhot City, East Ujimqin Banner, Abag Banner, Xianghuang Banner, the southern part of Siziwang Banner, and most areas of West Ujimqin Banner. The study area was located in Xilingol League and Siziwang Banner. These regions are severely affected by epidemics of grasshoppers, which are common occurrences in this area. Semi-arid is a specific climatic factor in the development of typical grasslands, with an annual precipitation of 250–450 mm and a humidity coefficient of 0.3–0.6 [30,31] (Figure 1). The landscape slopes from southeast to northwest, with mountains and low terrain in the southeast and flat terrain in the northwest. The dominant plants are *Stipa krylovii Roshev*., *Cleistogenes squarrosa*, and *Leymus chinensis* (Trin. ex Bunge) *Tzvelev* grasses [31]. The soil types mainly include meadow soils, chernozem, and kastanozem soils. Numerous potential pests inhabit the study area, including *Oedaleus decorus*, *Dasyhippus barbipes*, *Bryodema luctuosum*, and *Myrmeleotettix palpalis*. Despite various measures taken by the local government to control grasshopper outbreaks, grasshopper epidemics still affect half of Inner Mongolia, causing significant damage. The red triangular markers in Figure 1 represent locust sampling points.

### 2.2. Data Sources

#### 2.2.1. Characteristics of the *Oedaleus decorus asiaticus*

In Inner Mongolia’s Xilin Gol League, there are a total of 59 species of grasshoppers, among which *Oedaleus decorus asiaticus* holds a significant position, with a population proportion ranging from 50% to 60%, making it one of the main dominant species in Inner Mongolia’s grasslands [32]. *Oedaleus decorus asiaticus* belongs to the family Acrididae in the order Orthoptera. Its lifecycle is univoltine, with eggs overwintering in the soil. In late May to late July, the overwintered eggs hatch, reaching peak adult emergence from mid to late July. Adults choose sunny, exposed, compacted, and moist areas for oviposition from late July to early August. This species is ground-dwelling, preferring environments with higher ground temperatures, such as compacted sandy soils, sparse vegetation, and sunny slopes. They exhibit clear thermotaxis, being most active around midday and inactive during rainy or windy weather. *Oedaleus decorus asiaticus* has a wide distribution, from meadow grasslands in the east to typical grasslands in the central region, and even to desert grasslands in the central and western parts. This grasshopper mainly damages plants in the *Poaceae*, *Cyperaceae*, and *Iridaceae families*, particularly favoring grasses such as *Leymus chinensis*, *Stipa grandis*, *Cleistogenes squarrosa*, *Agropyron cristatum*, and *Carex* spp., as well as crops like maize, barley, and wheat [33,34,35,36].

#### 2.2.2. Data on Grasshoppers Occurrence 

Data on the distribution of grasshoppers were obtained from grassland stations affiliated with the Forestry and Grassland Bureau of Xilingol League, Inner Mongolia, which has monitored large-scale infestations of grasshoppers since early times and started to conduct systematic and standardized grasshopper monitoring after the Chinese Ministry of Agriculture issued specifications for grasshopper surveys in 2008, which were organized by the staff of the grassland stations. *Oedaleus decorus* field survey data from 2020 were obtained from the XilinGol Grassland Station and Siziwang Banner Grassland Station in Inner Mongolia, China. This study employed standard quadrat samplers to investigate the species and density of locusts. The specific procedure involved two individuals swiftly lowering the sampler to cover an area, allowing for the collection of relevant data such as locust species and quantity within the frame. The sampling of small individual locusts could also be conducted using quadrats with a side length of 50 cm, and data were recorded in terms of per-square-meter measurements during a statistical analysis. The grasshopper survey was conducted from June to August 2020, and the systematic survey observation plots were located in locust-prone areas, which could reflect the environmental characteristics of the region. Areas were selected where locust density at the outbreak level was observed for more than three years (including three years) within a ten-year period, and where, after control measures, the density remained at or below outbreak levels for three to five years. The pilot area covered approximately 100 hm^2^, with 10 plots selected within each pilot area. The distance between plots was no less than 1000 m. Finally, according to the national standard guideline for segmenting and monitoring inhabitable areas for locusts and grasshoppers in grasslands [37], when the density of grasshoppers exceeded 15/m^2^, the quadrat was marked as a grasshopper distribution point. Ten sample quadrats were randomly chosen with a minimum straight-line distance between quadrats of more than 100 m. Sites were marked as grasshopper distribution points when the grassland grasshopper density was >15/m^2^, and the staff also conducted grasshopper surveys in the vicinity of the annual outbreak areas, which were recorded in this dataset. We used these data to estimate the model.

Standard quadrat sampler: Construct frames measuring 1 m in length and 0.8 m in height, covered with mesh netting between the frames. Join every two frames along their short sides using hinges or other means, forming a semi-frame that can freely open and close to 90°. Two individuals each hold one half-frame, and when brought together, they form a four-sided enclosed square sampler.

#### 2.2.3. Remote Sensing Data 

A previous study by Lu identified that factors such as soil, vegetation, altitude, and temperature influenced the lifecycle and growth habits of grasshoppers [13]. Forty-two environmental variables related to grasshopper breeding were selected from bioclimatic environmental data. To simulate the suitability for grasshopper occurrence, four classes of environmental variables were considered, namely, topographical, meteorological, vegetational, and soil indicators.

The study used the MODIS product data of MOD11A2 from 2019 to 2020 to represent land surface temperature (LST), respectively. The MOD13A2 data for 2019–2022 was also used to represent the normalized difference vegetation index (NDVI) Both datasets had a spatial resolution of 1 km. The temporal resolution of the NDVI was 16 days, while the LST had a temporal resolution of 8 days. Maximum synthesis was used to obtain 16-day LST data. The FVC, or vegetation fraction coverage, was determined from the NDVI data as follows:

$$FVC=(NDVI-{NDVI}_{0})/({NDVI}_{v}-{NDVI}_{0})$$

where *FVC* indicates the vegetation coverage with pixel values ranging from 0 to 1, NDVI represents the normalized difference vegetation index, and ${NDVI}_{v}$ and ${NDVI}_{0}$ represent the values of pure vegetation and pure soil pixels, respectively.

#### 2.2.4. Meteorological Data

The China meteorological data-sharing network (http://www.nmic.cn/, viewed on 11 March 2022) provided meteorological data, such as monthly average temperatures and monthly cumulative precipitation. To obtain raster data with a spatial resolution of 1 km, kriging interpolation was conducted in Python. 

#### 2.2.5. Other Data

In general, there was little change in vegetation type, soil type, soil salinity, and elevation over short periods of time. Thus, these variables were assumed to undergo no significant changes during the study period. Geospatial data were obtained from the Chinese Academy of Sciences Geospatial Data Cloud. A national database with a resolution of 1:1,000,000 was used to obtain data on vegetation type and soil type. The digital elevation map had a spatial resolution of 30 m. All data were resampled to a spatial resolution of 1 km after pre-processing, including mosaic and projection conversion. Digital elevation data were used to calculate the aspect and slope.

### 2.3. Variable Filtering

**Figure 2:**
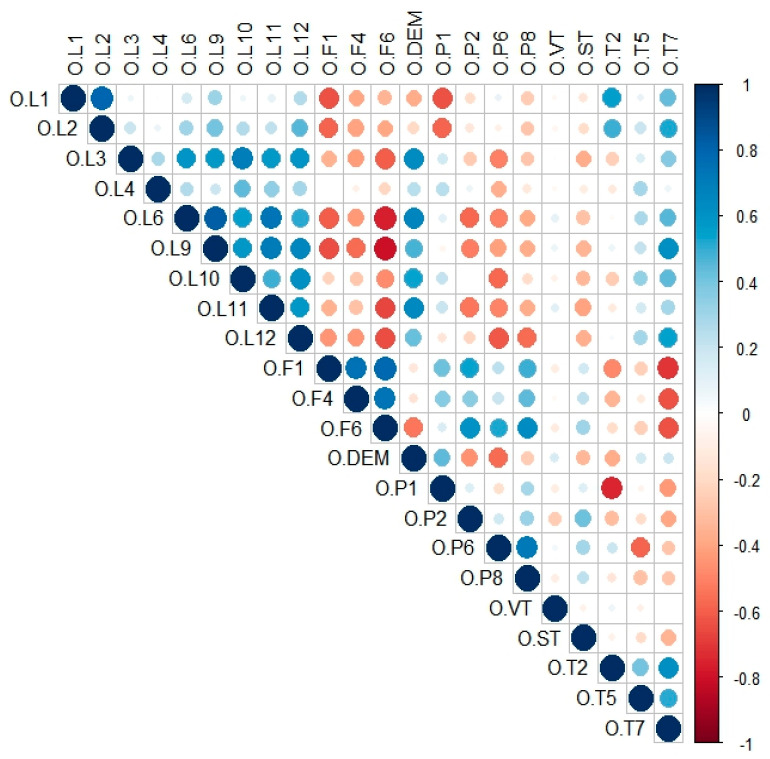
Distribution of correlation coefficients between habitat factors. The darker the blue, the higher the two environmental factors; the opposite is true for red.

To avoid multicollinearity among predictor variables and to accurately analyze the relationship between the species distribution and the environment, it was necessary to eliminate some causal variables that did not statistically contribute to the variation in the response variable. To create a high-performance model with fewer variables, cross-correlations (Pearson correlation coefficient, r) were determined among the variables. Only one variable from each set of highly cross-correlated variables (r > 0.8) was retained for further study based on the correlation analyses. For the *O. asiaticus* prediction, 22 variables were included(Table 1), namely O.L1, O.L2, O.L3, O.L4, O.L6, O.L9, O.L10, O.L11, O.L12, O.F1, O.F4, O.F6, O.DEM, O.P1, O.P2, O.P6, O.P8, O.VT, O.ST, O.T2, O.T5, and O.T7 (Figure 2).

### 2.4. MaxEnt Model and Evaluation

MaxEnt Version 3.4.1 (https://biodiversityinformatics.amnh.org/open_source/maxent/ accessed on 7 July 2021) was utilized to model the distribution of *O. asiaticus* in the typical steppe regions of central and eastern Inner Mongolia. MaxEnt is a versatile machine learning model that employs a precise and straightforward mathematical formulation. It is considered a presence-only model that utilizes predictor datasets to distinguish patterns in species occurrence.

(1) $$P_{w}\left(y|x\right)=\frac{1}{Z_{w}\left(x\right)}\exp\left(\sum_{i=1}^{n}w_{i}f_{i}\left(x,y\right)\right)$$

(2) $$Z_{w}\left(x\right)=\sum_{y}exp\left(\sum_{i=1}^{n}w_{i}f_{i}\left(x,y\right)\right)$$

where x represents each environmental variable input, y denotes the locations of grasshopper occurrence, $f_{i}\left(x,y\right)$ is the characteristic function, $w_{i}$ is the weight of the characteristic function, n represents the number of datasets, and $P_{w}\left(y|x\right)$ is the output of the spatial distribution of grasshopper occurrence in two grass types.

The predictive accuracy of the MaxEnt model was influenced by the optimization of two key parameters: the regularization multiplier (RM) and the Feature Combination (FC). The RM parameter modulates the concentration level of the output distribution. Higher values of the RM lead to a broader utilization in predictions, whereas lower RM values result in more concentrated output distributions, better suited to specific distributional records but prone to overfitting, thereby hindering model transferability to novel environments. The FC parameter, derived from variable climate layers, influences probability distribution calculations, with available feature types including linear (L), quadratic (Q), product (P), threshold (T), and hinge (H). In this study, model optimization was conducted using the R language kuenm package. Following the methodology of Cobos et al., the RM parameter varied from 0.1 to 4 in increments of 0.1, and 29 potential combinations of the five feature classes were selected. Subsequently, 2108 candidate models were generated using the kuenm_cal function, and the kuenm_ceval function was employed to identify the optimal combination of the RM and FC.

The MaxEnt model was configured with the following settings: a random test percentage of 25%, indicating that 25% of the presence localities were randomly withheld for testing to compute metrics such as areas under the curve (AUC) and omission; and a regularization multiplier of 1, signifying that all automatic regularization parameters were multiplied by this value, leading to a more dispersed distribution with higher numbers. The maximum number of background points was set to 10,000. The simulations comprised 100 replicates to determine mean relative occurrence or suitability probabilities. The output format was specified as logistic, and a jackknife method was applied to assess the importance of each environmental variable, whereby the model was trained with each variable first omitted, then used in isolation.

The MaxEnt model assumed a species equiprobability of occurrence across the landscape. The model performance was evaluated using the area under the receiver operating characteristic (ROC) curve (AUC). AUC values served as an independent threshold metric, ranging from 0 to 1, with values <0.5 indicating random prediction, 0.5 to 0.7 suggesting poor performance, 0.7 to 0.9 indicating moderate performance, and >0.9 indicating high performance.

## 3. Results

### 3.1. Modelling Optimization and Model Performance for Potential Distribution and Environmental Variables

**Figure 3:**
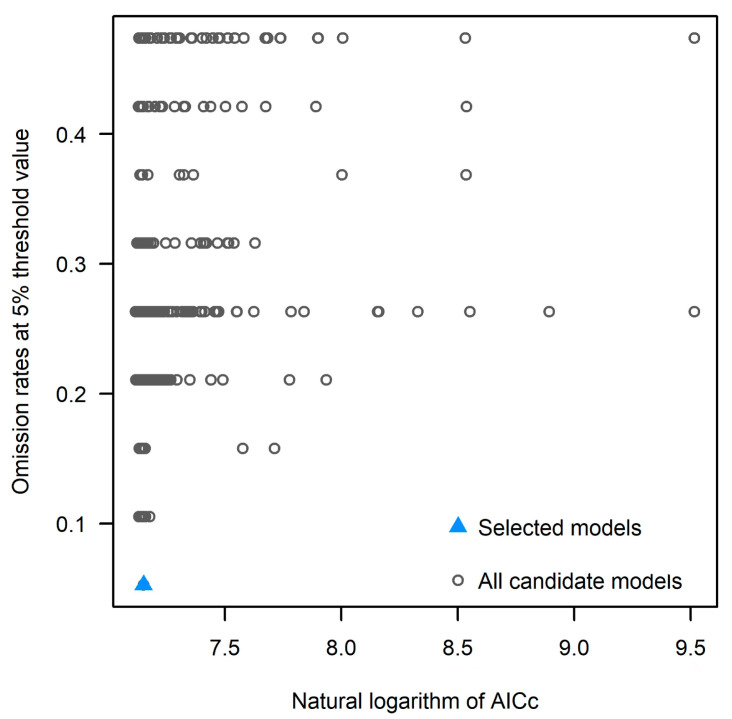
All candidate model data and the best model selected based on statistical significance, omission rate, and AICc criteria.

The kuenm package analysis showed that 1052 out of 1054 models of *O. asiaticus* were statistically significant, and 1 optimal model was selected from each of them based on the AUC value, the omission rate of less than 8%, and the minimum AICc value (as shown in Figure 3).

**Figure 4:**
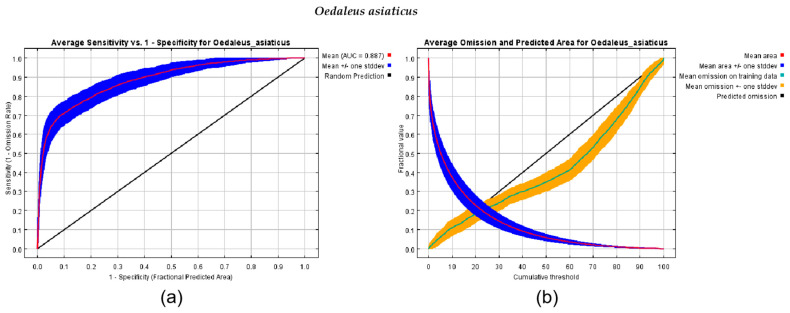
MaxEnt models for the typical central and eastern Inner Mongolian steppe regions represented by ROC curves and omission curves. (**a**) The ROC curve comprises a red line, a blue line, and an area under the curve (AUC) value. (**b**) The crimson line represents the model’s fit to the training data, while the blue line represents the model’s fit to the test data. The omission curve shows the differences between testing and training omission and the predicted area in relation to the cumulative threshold choice. Suitable conditions were predicted above the threshold value while unsuitable conditions were predicted below it. The omission rate should ideally be close to the predicted omission, according to the cumulative threshold definition.

The MaxEnt models utilized in this study exhibited favorable predictive accuracy, with AUC values surpassing 0.80, indicating a superior performance in predicting the habitat suitability of the *Oedaleus asiaticus* grasshopper species compared to random models. Specifically, the MaxEnt model resulted in a mean training AUC value of 0.887 for *Oedaleus asiaticus*. Validation based on 25% of the investigated grasshopper occurrence data demonstrated the modeling accuracy for predicting grasshopper occurrence in typical steppes. The omission curves for the typical steppes (Figure 4) showed that the mean omission on the test samples aligned closely with the predicted omission (the black straight line), indicating that there was a good fit between the model and the training data and that the test and training data were independent. The AUC curve indicated that in the central and eastern steppe regions, the simulated distribution of grasshopper occurrence was consistent with the collected data, with a value greater than that of the random model (0.5). 

### 3.2. Spatial Distribution of Grasshopper Occurrence in Typical Central and Eastern Inner Mongolian Steppes

**Figure 5:**
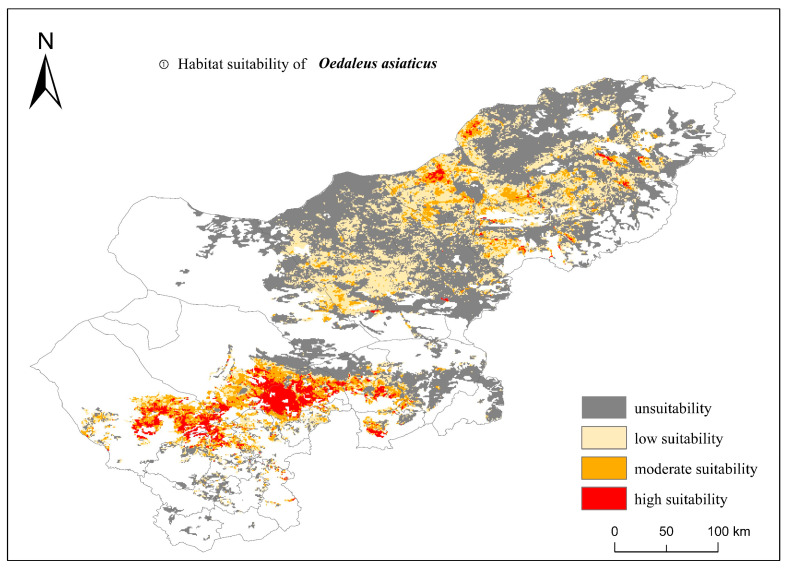
Modeling suitability of grasshopper occurrence in the typical central and eastern Inner Mongolian steppes determined by MaxEnt models. Four levels of suitability are shown, namely, high suitability (>0.75, red color), moderate suitability (0.5–0.75, orange color), low suitability (0.25–0.5, yellow color), and unsuitability (<0.25, gray color).

The association between habitat elements and grasshopper distribution was recreated using the MaxEnt model with the sample dataset and the combination of habitat factors influencing the grasshopper distribution. The probability of grasshopper occurrence at each grid point was assessed according to professional experience, with P < 0.25 considered an unsuitable area, 0.25 < P < 0.5 as a low-suitability area, 0.5 < P < 0.75 as a moderate-suitability area, and P > 0.75 as a high-suitability area. This showed that the unsuitable and low-, medium-, and high-suitability zones for grasshopper occurrence in the central–eastern grassland region were 69,116.4 km^2^, 34,767.11 km^2^, 13,946.43 km^2^, and 6138.304 km^2^, respectively, occupying 55.76%, 28.04%, 11.25%, and 4.95% of the grassland area (Figure 5).

### 3.3. Key Factors in Grasshopper Occurrence in Typical Central and Eastern Inner Mongolian Steppes

**Figure 6:**
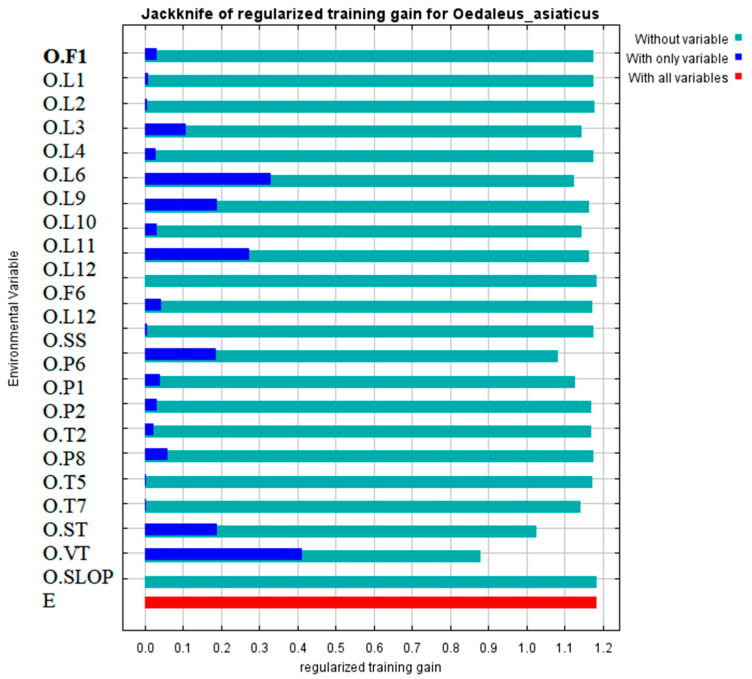
Jackknife of regularized training for the MaxEnt model of *Oedaleus asiaticus* occurrence in the typical central and eastern Inner Mongolian steppes.

**Figure 7:**
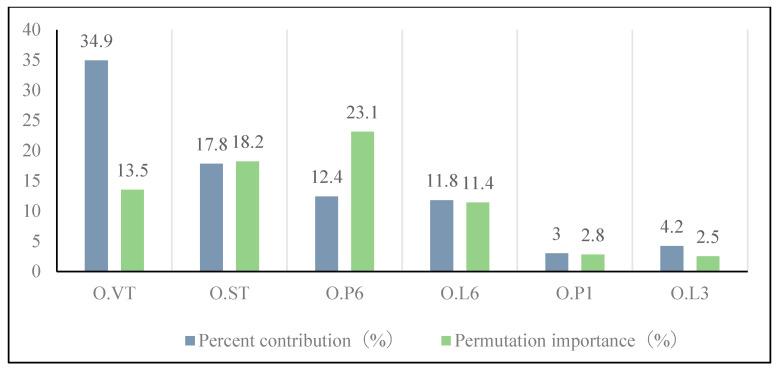
Percentage Contribution and Permutation Importance of environment variables for *O. asiaticus* in the MaxEnt.

The contribution rate represented the contribution of each habitat factor to the model; the higher the value, the greater the contribution of the element to the model. The random replacement of environmental elements at the training sample point was referred to as exchange importance. A larger reduction value, as well as a higher percentage of substitution, increased the model’s reliance on this variable. The results obtained from the jackknife test of variable importance (Figure 6) suggested that O.VT was the variable with the greatest gain when used in isolation. Furthermore, the omission of this variable resulted in the most significant decrease in the gain of the model value. The habitat components with the largest contribution rates, according to the jackknife approach, were O.VT (34.9%), O.ST (17.8%), O.P6 (12.4%), O.L6 (11.8%), O.P1 (3%), and O.L3 (2.1%) (Figure 7). Given the significance of replacement, six habitat factors, O.VT (vegetation type), O.ST (soil type), O.P6 (2019.12. precipitation), O.L6 (2019.12 land surface temperature) O.P1 (2019.7 precipitation), and O.L3 (2019.9 land surface temperature) were selected as the primary impact factors.

**Figure 8:**
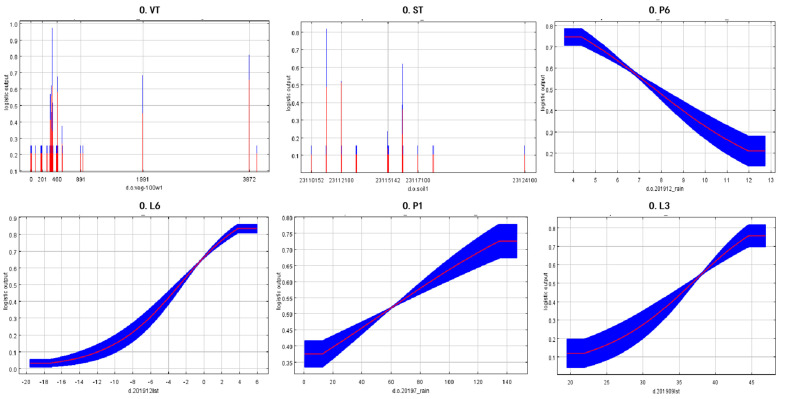
Response curves for the major habitat factors in the model predictions. The response curves show the relationship between the probability of grasshopper occurrence and habitat factors. The values shown are the mean of 100 replicate runs; blue edges show the ± SD of 100 replicates. For each panel, the x-axis represents the variable, and the y-axis represents the probability of grasshopper occurrence, Blue represents the standard deviation, while red represents the mean value.

The best model fit was obtained using the variables O.ST, O.L6, O.P1, O.P6, O.L3, and O.VT. These six habitat variables led to the greatest gains from independent training. Figure 8 shows the response curves for the seven habitat factors. The response curve of the model indicated that kastanozem, gray forest soil, and fluvo-aquic soils were the most suitable soil types for grasshoppers. The overwintering period (November 2019 to March 2020) is critically important for the Mongolian grasshopper, during which excessively low environmental temperatures can result in the significant mortality of grasshopper eggs, thereby impacting the scale of grasshopper outbreaks in the subsequent year. Throughout the overwintering period, the suitability of the habitat for grasshoppers exhibits a negative correlation with precipitation increase, with precipitation levels below 7.7 mm being conducive to grasshopper growth. Surface temperature is a pivotal environmental factor affecting grasshopper egg survival rates, with the suitability of the habitat positively correlating with rising surface temperatures; grasshopper suitability increases when surface temperatures exceed −3 degrees Celsius. During the growth period (June and July 2019), grasshopper suitability exhibits a positive correlation with increasing precipitation, being most favorable for grasshopper growth when precipitation levels are above 55 mm. In the breeding phase (August to September 2019), grasshopper suitability increases with rising surface temperatures. 

## 4. Discussion 

### 4.1. Evaluation of MaxEnt Output Performance

This study described the 2020 distribution of the grasshopper *Oedaleus asiaticus* using a machine learning model with a spatial resolution of 1 km in Inner Mongolia in China. Grasshoppers are pests that can devastate grasslands. The early detection of grasshopper outbreaks is critical. This study used three types of environmental variables, namely, geospatial, remote sensing, and meteorological data. Most previous studies have focused solely on the impact of meteorological factors on grasshopper occurrences in the Inner Mongolian grasslands [31]. Although meteorological factors are important in determining the suitability of grasshopper localities, the effects of vegetation and soil cannot be overlooked. The MaxEnt model and a set of optimized parameters were used to predict the potentially suitable distribution of grasshopper habitats in central and eastern Inner Mongolia. The results of the model showed that the distribution of suitable grasshopper habitats was determined by four major categories of habitat factors, namely, vegetation (O.VT), soil type (O.ST), precipitation (O.P), and land surface temperature (O.L). The simulations performed well, with a mean AUC of 0.887. In addition, the use of the MaxEnt model provided effective methodological support for monitoring grasshopper outbreaks on a large scale, while the accuracy of the model output can be further improved by selecting more environmental factors related to grasshopper occurrence in the future, with further research on the mechanism of grasshopper occurrence.

### 4.2. Potential Distribution of Oedaleus asiaticus 

The MaxEnt simulation demonstrated that the regions most suitable to be *Oedaleus asiaticus* habitats were primarily situated in the central and eastern areas of Xilin Gol, with moderate and high suitability primarily distributed in the eastern section of the study area. Conversely, regions of low suitability were concentrated in the northwestern and southwestern areas. Field surveys and literature reviews showed that Asian grasshoppers are mostly distributed in Siziwang Banner and Zhenglan Banner [32], as well as in Xianghuang Banner and significant numbers along the Xilin River [33,34]. All these locations were found within the predicted areas outlined in this study, indicating the reliability of the results.

### 4.3. Environmental Variables Affecting the Distribution of Oedaleus asiaticus 

Habitat selection by grasshoppers often depends on a complex combination of various interrelated environmental factors. It has been shown that soil characteristics (e.g., soil type, texture, temperature, moisture, pH) are important factors affecting grasshopper egg laying, egg hatching, and mortality, as well as adult populations and reproduction [35,38]. The results of this study showed that the most important environmental variable influencing the distribution of *Oedaleus asiaticus* was soil type (O.ST), confirming the key role of soil variables in the geographical distribution of *Oedaleus asiaticus*. Zhangna used fuzzy evaluation combined with 3S to evaluate the habitat suitability of grasshoppers and found that soil factors were the main environmental factors governing the geographical distribution of grasshoppers [39]. Mira L. Word [40] studied the relationship between land use, soil conditions, and locust abundance and concluded that desert locusts were most abundant in grazed and fallow areas and that improving soil fertility could be used as an alternative to insecticides to suppress locusts and improve crop yields. The results of the present study showed that the area of low suitability was situated mainly in the northwestern part of the study area, which contained the Hunshandake Sandland, where the main soil types are wind sand and brown calcareous soils with low soil nitrogen contents. At the same time, the structure of these sandy soils is loose and the water-retention capacity is poor, which is not conducive to the spawning and hatching of locusts. In the south, an area of high suitability is found in an extensive region of interlaced farming and animal husbandry areas [41], which are more suitable for locusts.

The temperature of the environment influences the physiological functioning of poikilothermic animals, such as insects. Temperature thus represents one of the most essential environmental factors next to photoperiod [42]. The jackknife analysis showed that the temperature variables associated with the distribution of *Oedaleus asiaticus* included O.L6 (overwintering temperature) and O.L3 (breeding temperature). The response curve illustrated in Figure 6 (O.L6) reveals an escalating suitability of locusts correlating with increasing surface temperatures during the overwintering period. Prior investigations, such as those conducted by Qi et al. [43], have demonstrated an elevation in the mortality rate of locust eggs under prolonged exposure to low temperatures. This phenomenon is attributed to the potential for excessively low temperatures to induce freezing or damage to locust eggs, thereby affecting their survival rate and consequently influencing the scale and severity of locust infestations in the subsequent year. Temperature significantly influences the growth cycle of locusts. Within the locust population, rates of foraging, locomotion, flight, dispersal, molting, mating, and oviposition decrease at lower temperatures and cease altogether at extremely low temperatures [44,45]. Generally, optimal temperature conditions facilitate successful reproduction in locusts. Our data suggested that with increasing surface temperatures, the suitability of locusts also steadily rises, implying that climate warming may impact the suitability and geographical distribution of typical grasshopper species by altering their lifecycle processes. Higher temperatures may also accelerate the maturation rate of locusts, thereby enhancing reproductive rates. Research by Xiongbing Tu et al. [46] indicated that during the reproductive phase of adult locusts, females from warmer environments often lay heavier egg masses. Additionally, adults tend to have longer lifespans, longer oviposition periods, and shorter oviposition intervals at higher temperatures. Therefore, temperature plays a crucial role in regulating the population size and distribution of locusts.

Plant communities are frequently influenced by fluctuations in precipitation, which can subsequently impact the vegetation associated with locusts in grasslands [47,48]. In previous studies, rainfall stress has been demonstrated to affect the life history of locusts and subsequently impact their reproduction [49]. In Lenhart’s research [50], increasing precipitation was shown to enhance the nutritional status of plant communities, while also confirming the potential negative impact of water scarcity on locusts. Concurrently, the augmentation of precipitation levels has elevated primary productivity, thereby more readily meeting the feeding requirements of locusts during their growth phase. The response curve for *Oedaleus asiaticus* during the breeding period (O.P1) showed that areas with precipitation above 55 mm were suitable. As one of the most important environmental factors, available water has a significant impact on the nutrient content of vegetation and the composition of plant communities. Precipitation indirectly affects the growth and reproduction of insects. In this study, our data showed that increased rainfall during the growth period had a significant impact on the suitability level; generally speaking, locusts require large amounts of energy during the breeding period, and precipitation will promote the growth of grassland vegetation, providing an adequate food source for the locusts.

The locusts found in Inner Mongolia’s temperate grasslands have a single generation per year, with the majority of their lifecycle spent in the form of eggs. As a result, the influence of climate change on eggs plays a critical role in determining the survival rate and hatchability of locust eggs. *Oedaleus asiaticus* is a species associated with dry conditions, so an increase in rainfall after egg laying has a greater negative effect on eggs, with excess rainfall increasing the probability of egg mold, and the higher the rainfall, the higher the probability of egg mold [50,51]. According to the response curve for the overwintering period (O.P6) depicted in Figure 6, suitability was higher between 2 and 3.8 mm of rainfall but declined to low levels with increasing rainfall. The eggshell structures of *O asiaticus* bear a striking resemblance to those of flying locusts, and both types of egg pods possess weakened and spongy crust structures [51,52]. Previous experiments have demonstrated that the water contents of the eggs of flying locusts vary with external conditions, and that *Oedaleus asiaticus* eggs are thus more sensitive to soil moisture. The shells of the egg pods have a greater buffering capacity against changes in soil moisture, and only prolonged exposure to low temperatures or high humidity causes significant mortality to locust eggs. This explains why the response curve did not decrease to very low levels; in winter, the locust eggs are already in a diapause state, and small fluctuations in rainfall do not have as significant an impact on locust eggs as they do in other seasons. 

To enhance the predictive accuracy of machine learning models, the selection of environmental factors is of paramount importance. Many studies have demonstrated that environmental factors exert a significant impact on the outcomes of these models. Here, we used Pearson’s correlation coefficient to assess the relationships between environmental variables, leading to the subsequent elimination of 12 highly autocorrelated variables.

### 4.4. Management Implications

To enhance the precision and efficacy of subsequent research, it is necessary to conduct more thorough and scientifically comprehensive ground surveys of grasshopper populations. *Oedaleus asiaticus* is the dominant grasshopper species in the grasslands of Inner Mongolia, with an extremely wide distribution. The insect spends nine months of the year in the underground stage (grasshopper eggs), and thus the hatchability of the eggs and survival of the hatched grasshoppers determine the size of the grasshopper outbreak in the following year. Future investigations could adopt this approach on a larger scale to assess the heterogeneity of *Oedaleus asiaticus* habitats across the region. Furthermore, the suitability of grasshopper breeding areas is constantly in flux due to global climate change, alterations in land use, and frequent overgrazing. Other marginally suitable areas and conditions could become suitable for grasshopper colonization in the near future, and thus environmental data should be monitored and updated to continuously refine current models [51].

## 5. Conclusions

This research examined the geographical distribution and significant environmental factors of *Oedaleus asiaticus* in the central and eastern regions of Inner Mongolia’s typical grasslands. Using a maximum entropy model, remote sensing, meteorological data, and geospatial information from a 2020 grasshopper ground survey, we investigated *Oedaleus asiaticus* in the grasshopper-prone area of Inner Mongolia and analyzed environmental factors associated with grasshopper outbreaks, such as weather, vegetation, soil type, and other variables. We screened 22 environmental factors that determine the suitability of *Oedaleus asiaticus* in typical grasslands. Meanwhile, special attention should be paid to the precipitation and temperature changes under specific vegetation types. Long-term monitoring of these changes can effectively monitor grasshopper activity. Our findings revealed that approximately one-third of the total area in central and eastern Inner Mongolia is conducive to *Oedaleus asiaticus*. The main focus lies in the central part of Ulanqab League, and the southern and northeastern parts of Xilin Gol League. These areas require strengthened monitoring and the long-term management of grasshoppers to mitigate the economic damage caused by their outbreaks. The soil type, reproductive period temperature, spawning period precipitation, overwintering period precipitation, and incubation period temperature are the critical factors determining *Oedaleus asiaticus* suitability in typical grasslands. Additionally, vegetation coverage plays a crucial role in the occurrence of *Oedaleus asiaticus*. All these environmental variables comprise the habitat preferences of *Oedaleus asiaticus*. Our study provides theoretical support for early grasshopper outbreak warnings, clarifies key *Oedaleus asiaticus* habitat factors in typical grasslands, and establishes a theoretical foundation for future grasshopper management.

## Figures and Tables

**Table 1:** The environmental factors used by the maximum entropy model. The table outlines the categories, factors, and descriptions. There are 22 factors in total.

Category	Factors		Description
LST	O.L1	201907LST	Land Surface Temperature of July in 2019
	O.L2	201908LST	Land Surface Temperature of August in 2019
	O.L3	201909LST	Land Surface Temperature of September in 2019
	O.L4	201910LST	Land Surface Temperature of October in 2019
	O.L6	201912LST	Land Surface Temperature of December in 2019
	O.L9	202003LST	Land Surface Temperature of March in 2020
	O.L10	202004LST	Land Surface Temperature of April in 2020
	O.L11	202005LST	Land Surface Temperature of May in 2020
	O.L12	202006LST	Land Surface Temperature of June in 2020
FVC	O.F1	201907fvc	Fraction of Vegetation Cover of July in 2019
	O.F4	201910fvc	Fraction of Vegetation Cover of October in 2019
	O.F6	202006fvc	Fraction of Vegetation Cover of June in 2020
DEM	O.DEM	DEM	Digital Elevation Model
Rain	O.P1	20197_rain	Precipitation of Wettest Month of July in 2019
	O.P2	20198_rain	Precipitation of Wettest Month of August in 2019
	O.P6	201912_rain	Precipitation of Wettest Month of December in 2019
	O.P8	20202_rain	Precipitation of Wettest Month of February in 2020
Veg	O.VT	veg	Vegetation Type
Soil Type	O.ST	Soil Type	Soil Type
Temp	O.T2	20198_temp	Average Temperature of August in 2019
	O.T5	20204_temp	Average Temperature of April in 2020
	O.T7	20206_temp	Average Temperature of June in 2020

## Data Availability

The data are not publicly available because the data need to be used in future work.
